# Pragmatic Trial Design to Compare Real-world Effectiveness of Different Treatments for Inflammatory Bowel Diseases: The PRACTICE-IBD European Consensus

**DOI:** 10.1093/ecco-jcc/jjae026

**Published:** 2024-02-17

**Authors:** Massimo Claudio Fantini, Gionata Fiorino, Agostino Colli, David Laharie, Alessandro Armuzzi, Flavio Andrea Caprioli, Javier P Gisbert, Julien Kirchgesner, Fabio Salvatore Macaluso, Fernando Magro, Subrata Ghosh, PRACTICE-IBD study group, Matthieu Allez, Aurelien Amiot, Raja Atreya, Manuel Barreiro-de Acosta, Livia Biancone, Fabiana Castiglione, María Chaparro, Axel Dignass, Glen Doherty, Eugeni Domènech, Walter Fries, Jonas Halfvarson, Uri Kopylov, Sara Onali, Daniela Pugliese, Davide Giuseppe Ribaldone, Simone Saibeni, Edoardo Vincenzo Savarino

**Affiliations:** Department of Medical Science and Public Health, University of Cagliari, Cagliari, Italy; Gastroenterology Unit, Azienda Ospedaliero-Universitaria di Cagliari,Italy; IBD Unit, Department of Gastroenterology and Digestive Endoscopy, San Camillo-Forlanini, Rome, Italy; Department of Gastroenterology and Digestive Endoscopy, San Raffaele Hospital and Vita-Salute San Raffaele Hospital, Milan, Italy; Department of Transfusion Medicine and Haematology, Fondazione IRCCS Ca’ Granda Ospedale Maggiore Policlinico di Milano, Milan, Italy; CHU de Bordeaux, Hôpital Haut-Lévêque, Service d’Hépato-gastroentérologie et Oncologie Digestive, Université de Bordeaux, Bordeaux, France; Department of Biomedical Sciences, Humanitas University, Pieve Emanuele, Milan, Italy; IBD Center, IRCCS Humanitas Research Hospital, Rozzano, Milan, Italy; Department of Pathophysiology and Transplantation, Università degli Studi di Milano, Milan, Italy; Gastroenterology and Endoscopy Unit, Fondazione IRCCS Cà Granda, Ospedale Maggiore Policlinico di Milano, Milan, Italy; Gastroenterology Unit, Hospital Universitario de La Princesa, Instituto de Investigación Sanitaria Princesa [IIS-Princesa], Universidad Autónoma de Madrid [UAM], Centro de Investigación Biomédica en Red de Enfermedades Hepáticas y Digestivas [CIBEREHD], Madrid, Spain; INSERM, Institut Pierre Louis d’Epidémiologie et de Santé Publique, Sorbonne Université, Department of Gastroenterology, Hôpital Saint-Antoine, Assistance Publique-Hôpitaux de Paris, Paris, France; IBD Unit, Villa Sofia-Cervello Hospital, Palermo, Italy; CINTESIS@RISE, Faculty of Medicine, University of Porto, Porto, Portugal; Department of Biomedicine, Unit of Pharmacology and Therapeutics, Faculty of Medicine, University of Porto, Porto, Portugal; Department of Clinical Pharmacology, São João University Hospital Center [CHUSJ], Porto, Portugal; Center for Health Technology and Services Research [CINTESIS], Porto, Portugal; College of Medicine and Health, University College Cork, Cork, Ireland; Service de Gastroentérologie, Hôpital Saint-Louis, APHP, Université Paris Cité, Paris, France; Department of Gastroenterology, Bicêtre University Hospital, AP-HP, Paris-Saclay University, Le Kremlin Bicêtre, France; Medical Department 1, University Hospital Erlangen, Friedrich-Alexander-University of Erlangen-Nürnberg, Ulmenweg, Erlangen, Germany; Gastroenterology Department of Complexo Hospitalario Universitario de Santiago, Santiago de Compostela, A Coruña, Spain; Department of Systems Medicine, University “Tor Vergata” of Rome, Rome, Italy; Gastroenterology, Department of Clinical Medicine and Surgery, University Federico II of Naples, Naples, Italy; Gastroenterology Unit, Hospital Universitario de La Princesa, Instituto de Investigación Sanitaria Princesa [IIS-Princesa], Universidad Autónoma de Madrid [UAM], Centro de Investigación Biomédica en Red de Enfermedades Hepáticas y Digestivas [CIBEREHD], Madrid, Spain; Department of Medicine I, Agaplesion Markus Hospital, Goethe University, Frankfurt, Germany; Department of Gastroenterology, St Vincent’s University Hospital and School of Medicine, University College Dublin, Ireland; Gastroenterology Department, Hospital Universitari Germans Trias i Pujol, Badalona; Centro de Investigación Biomédica en Red de Enfermedades Hepáticas y Digestivas—CIBEREHD; Departament de Medicina, Universitat Autònoma de Barcelona [Spain]; IBD-Unit, Department of Clinical and Experimental Medicine, University of Messina, Messina, Italy; Department of Gastroenterology, Faculty of Medicine and Health, Örebro University, Örebro, Sweden; Sheba Medical Center, Ramat Gan and Tel Aviv University Medical School, Tel AvivIsrael; Department of Medical Science and Public Health, Gastroenterology Unit, University of Cagliari, Cagliari, Italy; Gastroenterology Unit, Azienda Ospedaliero-Universitaria [AOU] di Cagliari, Cagliari, Italy; CEMAD, IBD Unit, Dipartimento di Scienze Mediche e Chirurgiche, Fondazione Policlinico Universitario “A. Gemelli” IRCCS, Università Cattolica del Sacro Cuore, Rome, Italy; Dipartimento di Scienze Mediche, Università degli Studi di Torino, Turin, Italy; Gastroenterology Unit, Rho Hospital, ASST Rhodense, Rho [MI], Italy; Department of Surgery, Oncology and Gastroenterology, University of Padova, Padova, Italy

**Keywords:** Pragmatic clinical trials, inflammatory bowel diseases, consensus procedure

## Abstract

**Background and Aims:**

Pragmatic studies designed to test interventions in everyday clinical settings can successfully complement the evidence from registration and explanatory clinical trials. The European consensus project PRACTICE-IBD was developed to identify essential criteria and address key methodological issues needed to design valid, comparative, pragmatic studies in inflammatory bowel diseases [BDs].

**Methods:**

Statements were issued by a panel of 11 European experts in IBD management and trial methodology, on four main topics: [I] study design; [II] eligibility, recruitment and organisation, flexibility; [III] outcomes; [IV] analysis. The consensus process followed a modified Delphi approach, involving two rounds of assessment and rating of the level of agreement [1 to 9; cut-off ≥7 for approval] with the statements by 18 additional European experts in IBD.

**Results:**

At the first voting round, 25 out of the 26 statements reached a mean score ≥7. Following the discussion that preceded the second round of voting, it was decided to eliminate two statements and to split one into two. At the second voting round, 25 final statements were approved: seven for study design; six for eligibility, recruitment and organisation, flexibility; eight for outcomes; and four for analysis.

**Conclusions:**

Pragmatic, randomised, clinical trials can address important questions in IBD clinical practice, and may provide complementary, high-level evidence, as long as they follow a methodologically rigorous approach. These 25 statements intend to offer practical guidance in the design of high-quality, pragmatic, clinical trials that can aid decision making in choosing a management strategy for IBDs.

## 1. Introduction

Inflammatory bowel diseases [IBD] include ulcerative colitis and Crohn’s disease. Both diseases may occur in adults and adolescents and affect men and women equally.^1^ Chronic inflammation in IBD seems to be triggered and maintained by the interaction between genetic and environmental factors which influence mucosal immune response against the intestinal microbiota. Over the past decade, there has been rapid evolution of medications and treatment strategies for the management of IBD. Basic and translational research has shed light on the mechanisms involved in the pathogenesis of IBD, leading to the identification of molecules and pivotal immune pathways, which are now the targets of some of the most innovative therapies available in the field.^2^ Although new biologics and small molecules have shown significant efficacy compared with placebo, disease control in randomised clinical trials [RCTs] is achieved by no more than 30–40% of patients, depending on the outcome considered.^3–5^

RCTs have long been considered the gold standard of clinical research.^6^ Nevertheless, high cost, organisation burden, long time to completion, use of placebo [which is not an alternative in real-world settings], restricted selection of participants and therapeutic approach and, last but not least, the application of patient-management protocols not in line with clinical practice, are considered major flaws of this methodological approach. These limitations make data from RCTs poorly transferable to real-world clinical practice. Furthermore, head-to-head RCTs directly comparing treatments are often not available and evidence for drug positioning is missing. On the other hand, most real-world efficacy data on newly introduced IBD therapies come from retrospective studies^7,8^ or prospective registries,^9,10^ which however may produce low-quality evidence because of the high risk of biases. In retrospective studies, particularly when multiple centres are involved, recall bias and unstandardised practices might generate unreliable results. Prospective registries can undoubtfully provide data from large patient cohorts, overcoming recall biases and ensuring some level of standardisation in data collection. However, the data available from registries are predefined and may not be suitable for investigating specific outcomes. Such a scenario leaves a gap of knowledge in between. This gap is particularly evident when considering real-world data on comparative effectiveness to define drug positioning in clinical practice.

Pragmatic trials are designed to test effectiveness of interventions in real-life clinical practice settings, to maximise applicability and generalisability of outcomes by modifying routine care minimally or not at all. The pragmatic intent generally favours the choice of major outcomes, such as mortality and severe morbidity, which are tested in a broad range of participants. In this way, pragmatic trials answer the question of whether an intervention works in the real world. Pragmatic trials may include complex interventions and several interacting components, and may involve more than one health care professional to deliver the intervention.^11^ Pragmatic trials were first proposed by Schwartz and Lellouch in 1967 as opposed to explanatory trials, in the sense that the latter are designed to confirm a physiological or clinical hypothesis set in optimal conditions, whereas the former are designed to inform a clinical or policy decision by providing evidence for adoption of the intervention into real-world clinical practice.^12,13^ More recently, the PRECIS and then PRECIS-2 tools were developed to help trialists make design decisions consistent with the intended purpose of their trial, with nine domains—eligibility criteria, recruitment, setting, organisation, flexibility in delivery, flexibility in adherence, follow-up, primary outcome, and primary analysis—scored from 1 [very explanatory] to 5 [very pragmatic].^14,15^ An extension of the CONSORT statement has been produced specifically to improve the reporting of pragmatic studies^16^ and, in more recent years, a thorough analysis of their possible ethical and regulatory issues has also been performed.^17^ Therefore, pragmatic trials may overcome the limitations of placebo-controlled or regulatory RCTs for application in routine clinical practice conditions.

In the IBD. pragmatic trials comparing the effectiveness of IBD treatments in the real world are rare and pragmatism poorly defined. One example of pragmatic trial in IBD is represented by the CONTRUCT trial. This trial was designed to compare the efficacy of ciclosporin and infliximab in the context of acute severe ulcerative colitis [ASUC] as it would be managed in a real-world setting.^18^ More recently, another pragmatic trial explored the noninferiority of disease activity-guided adalimumab interval £lengthening compared with standard dosing of every other week [EOW] in patients affected by Crohn’s disease [CD]. In both the cases, the definition of pragmatic was self-defined and not based on established criteria.^19^ The European consensus project PRACTICE-IBD was implemented to foster expert discussion on how to address this issue and reach a consensus. The work focused on the identification of minimum criteria to design a pragmatic study following the PRECIS-2 domains,^15^ and on the production of statements to guide the appropriate design of comparative pragmatic studies in IBD.

## 2. Methods

Consensus participants were recruited through the network of national IBD groups, according to their experience in the field of IBD and in real-world studies. Originally, the project started as an Italian national initiative, but as a result of meetings and discussions with international experts it was decided to extend the consensus to other countries with similar characteristics to Italy.

The consensus process followed a modified Delphi approach, consisting of several steps of assessing and rating the level of agreement with recommendations issued by a panel of experts. The methodology was adopted to be in accordance with other consensus work conducted in the field of IBD. The modified Delphi approach is an established method of reaching a consensus opinion among a group of experts in a particular field, and is also used to produce best practice guidelines.^20,21^ Given the international composition of the consensus group, all the meetings were virtual to facilitate organisation and ensure feasibility.

**Figure 1:**
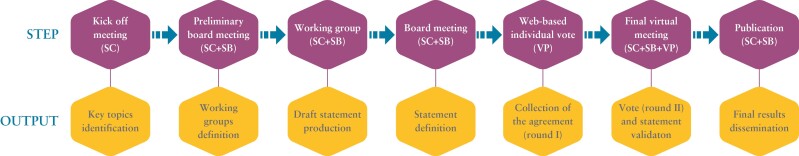
Graphic representation of steps and outputs leading to the development of consensus statements. SC, Steering Commitee [*N* = 5]; SB, Scientific Board [*N* = 6]; VP, Voting Panel [*N* = 18].

The Steering Committee [SC] was composed of five members [MCF, GF, and AC from Italy, SG from Ireland, and DL from France], with expertise in managing adult IBD and in trial methodology, previous involvement in IBD clinical trials, and scientific publications in the field. At its first meeting, on November 17, 2022, the SC reviewed the most recent literature on pragmatic trials in general and IBD in particular. A detailed search strategy is available in the Supplementary material [Supplementary Methods and Table S1]. The experts agreed to issue recommendations on the following four topics covering the PRECIS-2 domains^15^: [I] study design; [II] eligibility, recruitment and organisation, flexibility; [III] outcomes; [IV] analysis. On December 19, 2022, the SC met with a larger Scientific Board [SB] composed of six or more experts in IBD and trial methodology [AA, FAC, FSM from Italy, JK from France, JPG from Spain, and FM from Portugal] to discuss the literature review and the selected topics. They split into four working groups, each of which was invited to meet virtually during the following 2 months to discuss and draft 6–7 statements each, with guidance from a list of questions prepared for each topic by the SC. The proposed statements were then discussed and finalised at the second SC + SB joint meeting which was held on February 1, 2023. Overall, 26 statements were produced and agreed upon. During March and April 2023, consensus on these statements was sought by additional European IBD experts. Experts were recruited based on scientific profile, experience in conducting clinical trials, role in scientific societies, and experience in clinical consensus panels. The first round of voting was web-based: the experts who confirmed their interest in participating were provided with a link to access an online landing page to vote on the statements. Access was restricted to authorised and registered persons only. Further information on the procedure for recruiting voting experts can be found in the Supplementary material [Figure S1]. The involved clinicians were asked to rate their level of agreement with the 26 proposed statements using a 9-point numerical rating scale [1 = do not agree at all, 9 = agree completely]. Voting was conducted in a blinded fashion and the results were collected as aggregated data. Upon receipt, first-round votes were analysed, and mean values were calculated. On May 19, 2023, a final virtual meeting was set up between the SC, the SB, and 18 voting experts [Figure 1]. The statements were presented together with the results of the first voting round and discussed thereafter. Each participant was free to contribute to the discussion, taking the floor and suggesting any changes or re-evaluations. If changes to the text were agreed during the discussion, the statements were amended in real time accordingly before undergoing a second round of direct voting on the same 9-point numerical rating scale. The second round of voting was conducted in a blinded fashion and exclusively by the 18 voting experts. The statements reaching an average score ≥7 at the second voting round were considered approved, whereas the statements that could not reach a mean score ≥7, despite discussion and amendments, were considered rejected.

## 3. Results

At the first voting round, 25 out of the 26 statements reached a mean score ≥7. Only one scored <7 [mean score 6.9]. Following the discussion that preceded the second round of voting, it was decided to eliminate two statements and to split one into two. For all statements, the mean level of agreement improved at the second voting round, so that all were considered approved. The complete list of statements with the results of the first and second round of voting and the changes made is shown in Table S2 of the Supplementary materials. Table 1 summarises the 25 final statements, which are reported with their final mean level of agreement and discussed hereafter.

**Table 1:** Final consensus statements agreed on how to design a clinical trial in IBD.

Topic 1—Study design	
1	Randomisation should be considered as the first option to assess and compare the effect of interventions in a pragmatic trial.
2	The unit of randomisation and analysis may be patients, but also health care practitioners, communities, or health care institutions such as clinics, depending on the nature of the intervention, setting and aim of the study.
3	Cohort multiple randomised controlled trials [cmRCTs] allow effective recruitment and retention of patients and make trials efficient and patient-centred.
4	Adaptive trial design may increase efficiency and make trials more conclusive, especially if multiple interventions are compared at the same time.
5	Longitudinal [follow-up] cohort studies should be preferably prospective but can also use a retrospective design.
6	To identify possible modifiers of effects, planning prespecified subgroup analyses is recommended.
7	Noninferiority trials may be justified if the investigated therapy is cheaper, more convenient, or safer than the control therapy.

Topic 2—Eligibility, recruitment and organisation, flexibility	
8	Minimal criteria for eligibility in pragmatic studies aiming to compare drug effectiveness should include confirmed diagnosis and indication to receive the drugs under investigation, according to Summary of Product Characteristics [SmPC] or robust scientific evidence.
9	In prospective studies, consecutive patients with the diagnosis of interest and indication for the treatment under study accessing participating centres should be invited to participate.
10	Recruitment should be performed in the context of standard clinical practice.
11	Patients should be treated in a real-world setting, according to local clinical practice.
12	The intervention should be given as in clinical practice, and dosing regimens should be clearly defined in the study protocol and rigorously reported.
13	A pragmatic study design should include minimal restrictions on concomitant therapies, to reflect practical setting.

Topic 3—Outcomes	
14	In pragmatic studies comparing drugs or strategies, effectiveness can be defined as the capability of any intervention to achieve predetermined endpoints relevant for producing a desired result in clinical practice.
15	Depending on the question of interest, the results should be assessed considering the perspectives of the physician, the patient, and/or the health care system.
16	Objective established measures of effectiveness should be preferred to assess the primary outcome.
17	A pragmatic trial should have only one primary endpoint. A composite primary endpoint may also be considered.
18	Outcome measures should always be clearly defined in a pragmatic trial; they should be consistent, as much as possible, with those used in similar trials.
19	Outcomes should be assessed after an adequate time interval depending on the drug/strategy investigated.
20	Safety should be assessed considering any adverse event possibly related to the study drug/intervention. Serious adverse events and adverse events leading to discontinuation should always be recorded and analysed separately.
21	If relevant to an intervention, sample size should be adequate to detect uncommon adverse effects of interest.

Topic 4—Analysis	
22	As the intention-to-treat effect is the effect of interest in pragmatic trials, the primary analysis should be performed according to the intention-to-treat approach. A per-protocol analysis may be considered as part of secondary analyses.
23	In a pragmatic trial, the planned methods to deal with missing data should be prespecified in the protocol.
24	Complex statistical methods to handle missing data, such as multiple imputation or covariate adjustment, are to be preferred to single imputation methods.
25	The emulation of a hypothetical target trial using non-randomised, real-world data reduces the risk of self-inflicted bias. Appropriate propensity score methods may achieve balance between groups on collected variables.

## 4. Discussion

### 4.1. Topic 1—Study design

#### 4.1.1. Statement 1 [mean score: 8.6]

##### Randomisation should be considered as the first option to assess and compare the effect of interventions in a pragmatic trial.

A comparison study including a control group increases the strength of the evidence. Furthermore, in order to obtain a reliable assessment of the effects [intended effects] of the investigated treatment, concealed randomisation is needed. In non-randomised studies, confounding by indication is virtually unavoidable due to a link between prognosis and prescription. Only randomisation and allocation concealment to treatment ensure that patient groups are balanced with respect to both known and unknown confounders and that the assessment of the effects of the treatment is unbiased.^6^

Conducting a randomised controlled trial, even pragmatic, may be quite expensive. This may be a relevant limitation, particularly for independent research. However, compared with explanatory RCTs on medications, costs should be sustainable since drugs and procedures are given and conducted as part of the standard of care. Moroeover, the costs for randomisation, data monitoring, and data collection on validated electronic case report forms, may be reduced if scientific societies or similar entities [especially public entities or patients’ associations] would cover these costs by research grants or other similar initiatives. This aspect was also considered when we set up this consensus, in order to gather together experts from countries with similar health care and independent research support systems.

#### 4.1.2. Statement 2 [8.2]

##### The unit of randomisation and analysis may be patients, but also health care practitioners, communities, or health care institutions such as clinics, depending on the nature of the intervention, setting, and aim of the study.

Clinical trials most frequently involve patients, but in some cases the participants may be groups of practitioners or the health systems when the study aims at improving some aspect of care. In cluster-randomised trials [CRTs], groups of individuals rather than individuals are randomised to different interventions. The ‘unit of allocation’ is the cluster or the group. The groups may be, for example, schools, villages, medical practices in hospitals, clinics, physicians’ practices, or families. CRTs may be done for several reasons. One may be to evaluate the group effect of an intervention. For example, CRTs might be chosen when the target of the intervention is health professionals with the aim of assessing their impact on patient outcomes, or a collective or system rather than individual patients. A CRT is better suited to evaluate whether a new standard of care, guideline recommendation, or other practice, hospital, or system level change, is affecting patient outcomes compared with existing standard of care.^22^

#### 4.1.3. Statement 3 [8.5]

##### Cohort multiple randomised controlled trials [cmRCTs] allow effective recruitment and retention of patients making trials efficient and patient-centred.

The cmRCTs design is a new approach primarily intended for pragmatic, randomised, controlled trials [pRCTs], to address their limitations by aligning them more closely to actual health care practice. This innovative trial design is increasingly used because of its efficiency in recruitment, advantages in reducing subject burden, and ability to better mimic real-world consent processes.^23,24^ In fact, novel interventions are trialled within much larger, typically longitudinal, cohorts of patients. First, all participants in the cohort who are eligible for treatment are identified; then, a random sample of patients is selected and are offered treatment which they can either consent to receive or refuse. All remaining eligible patients [i.e. all patients eligible for treatment but not offered treatment] form the control arm. Relevant outcomes are assessed on all patients in both arms as part of the regular follow-up process. Further cmRCTs of other interventions can be conducted within the same core cohort of patients. All cohort patients give their informed consent to the use of observational data at the outset; however, consent to ‘test’ a particular intervention is sought only from those offered that intervention. Whereas the cmRCT has several advantages, some key methodological challenges to its use have been identified in actual practice which can threaten validity.^25^ In order not to compromise validity and to minimise potential bias, cmRCTs require tailored power calculation, sampling, and analysis procedures.

#### 4.1.4. Statement 4 [8.3]

##### Adaptive trial design may increase efficiency and make trials more conclusive, particularly if multiple interventions are compared at the same time.

In adaptive trials, interim [adaptive] analysis results are used to modify aspects of the trial, such as including adaptive stopping rules or sample sizes, adaptive arm drop, and adaptive response randomisation. This may lead to greater efficiency and more conclusive studies, particularly if multiple interventions are compared simultaneously or against the same control group, with the lower arms dropped early.^26^ Adaptive trials increase the likelihood of finding any benefit of the intervention being studied and help identify participants who are more likely to benefit from the intervention.^27^ However, these features also add to the complexity, requiring proper planning, specific methodological knowledge, extensive simulation-based comparison, and adequate resources for interim assessment/s.^28,29^

This methodology was successfully applied during the COVID19 pandemic by platform clinical trials networks such as RECOVERY and ACTIV, which intentionally integrated clinical research into clinical care.^30,31^

#### 4.1.5. Statement 5 [8.1]

##### Longitudinal [follow-up] cohort studies should preferably be prospective but can also use a retrospective design

The distinction between prospective and retrospective studies has been debated for a long time.^32^ Some experts consider studies derived from medical records strictly retrospective, whereas other consider any follow-up study to be prospective even if historical data are used. When researchers collect data according to a protocol and follow the included participants until specific disease or mortality endpoints are met, the collected data can be considered accurate for exposure, confounders, and endpoints. However, these benefits come at the expense of efficiency, due to the high costs and the complexity of a long follow-up. On the other hand, if a study starts at a time when follow-up has already been completed, existing data can provide useful information in a very time-efficient way; but there is no other choice but to analyse what has been measured in the past, often for a purpose other than that of the study. non-randomised, observational analyses of large electronic patient databases might be properly used to assess adverse events, specially the rare ones.^33^ non-randomised, real-world studies, with data that are fit for purpose and have used proper design and analysis, can also be useful to estimate and compare treatment effectiveness. We believe that, to evaluate and compare the effectiveness of interventions, it is more relevant to reduce the effect of possible confounders with adequate adjustment and minimisation of selection bias, losses to follow-up, or missing data. If a database contains all the information relevant to answering the clinical question, it can yield the same results, with the same value and strength and greater efficiency, than a specifically designed prospective study.^34–36^

#### 4.1.6. Statement 6 [8.2]

##### To identify possible modifiers of effects, planning prespecified subgroup analyses is recommended.

Planning prespecified subgroup analyses to identify possible moderators of effects allows for the trial design to be as simple as possible, and can reduce the need for stratification before randomisation which can make the design and the randomisation process more complex.^37^ Post hoc subgroup definition and analysis may carry the risk of multiplicity.^38^ Conversely, stratified randomisation prevents imbalance and ensures a similar distribution between treatment groups in important variables thought to influence outcome, such as age and disease stage. Stratification may prevent type I error and improve power for small trials. Stratified randomisation is important only for small trials in which treatment outcome may be affected by known clinical factors that have a large effect on prognosis, large trials when interim analyses are planned with small numbers of patients, and trials designed to show the equivalence of two therapies.^39^

#### 4.1.7. Statement 7 [8.4]

##### Noninferiority trials may be justified if the investigated therapy is cheaper, more convenient, or safer than the control therapy.

Noninferiority trials assess whether a new intervention is not less effective by a specified margin than a comparator standard treatment. Assessing noninferiority in a trial is more complex than assessing superiority, in both the design and analysis phases, since the definition of noninferiority margin is complex and requires analyses of previous RCTs results and is also somehow counterintuitive. However, the number of randomised trials assessing noninferiority has greatly increased in the past decade. Such studies should concern a new intervention that does not offer greater efficacy than standard treatment but may promise another clinically or financially relevant benefit, such as less intensive dosing, fewer side effects, greater administration convenience, or lower cost. If a new therapy offers no benefit on any of these parameters, testing it in a noninferiority study is unethical.^40–42^ In noninferiority trials, new treatment is evaluated just for efficacy similar to that of an established treatment, just to find out a good substitute. A proper noninferiority design requires: prior randomised trials evaluating the superiority of the active control over placebo; the definition of an acceptable noninferiority margin that cannot be greater than the smallest effect size for the active treatment that would be expected in a placebo-controlled trial; an appropriate metric likely to be constant between studies and therefore a reliable metric for comparison between-group difference [e.g. absolute risk or relative risk]; adequate trial execution and outcomes, ascertainment. Incomplete treatment adherence could bias results toward a conclusion of noninferiority. Either intention-to-treat or per-protocol analysis may produce biased and inconclusive results.^40,42^ A particular challenge concerning the safety assessment in noninferiority designs is that there are usually no reasonable data to justify the definition of margins for safety. More in general, there is evidence of poor reporting and conduct of noninferiority trials.^40^

### 4.2. Topic 2—Eligibility, recruitment and organisation, flexibility

#### Statement 8 [8.4]

##### Minimal criteria for eligibility in pragmatic studies aiming to compare drug effectiveness should include confirmed diagnosis and indication to receive the drugs under investigation according to Summary of Product Characteristics [SmPC] or robust scientific evidence.

Pragmatic studies require participants to have the condition of interest and be potential candidates for the study intervention in the usual care for this condition. Inclusion and exclusion criteria should be minimal.^11,17^ All participants who have the condition of interest should be enrolled, regardless of their anticipated risk, responsiveness, comorbidities, or past compliance.^16^

#### 4.2.2. Statement 9 [8.4]

##### In prospective studies, consecutive patients with the diagnosis of interest and indication for the treatment under study accessing participating centres should be invited to participate.

To avoid selection bias, it is mandatory to enrol all consecutive patients accessing the clinic or specified practice within the defined time frame who meet the eligibility criteria. Missing data should be handled as indicated below, in Statement 24.

#### 4.2.3. Statement 10 [8.8]

##### Recruitment should be performed in the context of standard clinical practice.

Recruiting patients in standard routine clinical practice is the most pragmatic approach. Recruiting study participants with the condition of interest from as many institutions as possible is even more pragmatic, since such a multicentre approach would increase the applicability of the study results.^16,43^ On the other hand, using recruitment methods that require activities and resources not normally present in standard clinical practice would reduce the trial pragmaticism, moving the trial more towards the explanatory side.^17^ One possible challenge may be the distribution of patient enrolled in each centre in the context of multicentre trials. Although well-balanced populations across participating text may increase homogeneity in the overall study population, this may be not required for pRCTs, as the difference between populations in each centre may highlight some important practical aspects of managing IBD patients, which could be of added value to increase external validity of the results.

#### 4.2.4. Statement 11 [8.6]

##### Patients should be treated in a real-world setting, according to local clinical practice.

In pragmatic trials, the intervention should be delivered as in clinical practice, by personnel usually involved in this activity and with the use of commonly available equipment. No trial-specific data collection visits should be planned outside the routinely scheduled visits for the condition under study. Treating patients in a real-world setting also means relying on local human resources commonly involved in the everyday practice.^11,17^ If necessary, extra staff specifically required for outcome evaluation might be allowed, provided this does not impact on patients’ management.

Although patients from a real-world setting may be quite heterogeneous in terms of baseline characteristics, we discussed and agree that this risk is overcome by the higher external validity given by the results coming from a real-world population in a pRCT compared with an explanatory RCT, which will keep the level of evidence still moderate to high compared with the low- or very low-quality evidence coming from pure observational real-world studies.

#### 4.2.5. Statement 12 [8.7]

##### The intervention should be given as in clinical practice, and dosing regimens should be clearly defined in the study protocol and rigorously reported.

If the intervention is a medical therapy, the standard dosing regimens should be specified in the protocol and any possible changes should be reported and justified. For other types of experimental interventions, instructions on how to apply them should be rather flexible, leaving practitioners considerable freedom.^16^

#### 4.2.6. Statement 13 [8.7]

##### A pragmatic study design should include minimal restrictions on concomitant therapies, to reflect practical setting.

For a study to score highly pragmatic, the protocol should neither dictate nor restrict concomitant interventions. No specific indications should be given for type and timing of concomitant therapies. If the intended comparator is standard care, introducing changes in its delivery is not compatible with a pragmatic design. In cases where the comparator is not standard care, treatment delivery flexibility should apply to all treatment arms to ensure a pragmatic approach.^11^

### 4.3. Topic 3—Outcomes

#### 4.3.1. Statement 14 [8.7]

##### In pragmatic studies comparing drugs or strategies, effectiveness can be defined as the capability of any intervention to achieve predetermined endpoints relevant for producing a desired result in clinical practice.

This is the standard definition of effectiveness. The concept of effectiveness applies to clinical practice in real-world conditions, as opposed to the concept of efficacy, which refers to ideal conditions as in RCTs.

#### 4.3.2. Statement 15 [8.1]

##### Depending on the question of interest, the results should be assessed considering the perspectives of the physician, the patient, and/or the health care system.

The study outcome[s] should be meaningful to study participants. This meaningfulness can refer, depending on the question of the study, for instance to clinical advantages for patients, practicality/convenience for clinicians and/or patients, or economic benefits for the health care system.

#### 4.3.3. Statement 16 [8.5]

##### Objective established measures of effectiveness should be preferred to assess the primary outcome.

Primary study outcomes dependent on the subjective investigator’s judgement should be avoided. Subjective measures have been shown to suffer from many systematic biases related to order, scale, halo-effects, and psychological factors. Furthermore, it has been shown that subjective measures are often uncorrelated with independent, objective measures related to the variable of interest. These objectives measures include at least biomarkers, but endoscopy and/or non-invasive techniques, such as bowel ultrasound, magnetic resonance, or computerised tomography enterography, should also be considered depending on the study setting.^44^ Subjective elements, if necessary, can be admitted in a rigorous clinical research methodology only if they can be accurately defined, measured, and represented.^45^

#### 4.3.4. Statement 17 [8.7]

##### A pragmatic trial should have only one primary endpoint. A composite primary endpoint may also be considered.

Recognising the importance of patient-reported outcomes [PROs] in IBDs, regulatory authorities have recommended also evaluating PROs in IBD clinical trials. These could represent co-primary endpoints in pragmatic trials, provided they are measured by standard scales/scores.^46^

#### 4.3.5. Statement 18 [8.6]

##### Outcome measures should always be clearly defined in a pragmatic trial; they should be consistent, as much as possible, with those used in similar trials.

The use of outcomes currently adopted in the literature, when available, may give the opportunity to compare different trials, increasing the reliability of the results, and the possibility to meta-analyse data to increase the level of evidence.

#### 4.3.6. Statement 19 [8.4]

##### Outcomes should be assessed after an adequate time interval, depending on the drug/strategy investigated.

Short-term response should be assessed at the latest time a drug is expected to exert its therapeutic effect, as this may be different from one drug to another. Long-term outcomes should be assessed not earlier than 52 weeks. Outcomes such as persistence in treatment, and rates of hospitalisation and surgery, should be avoided since they can be strongly influenced by local guidelines/drug availability/reimbursement policies.

#### 4.3.7. Statement 20 [8.0]

##### Safety should be assessed considering any adverse event possibly related to the study drug/intervention. Serious adverse events and adverse events leading to discontinuation should always be recorded and analysed separately.

Pragmatic studies provide safety data in unselected populations. However, such data are often self-reported, incomplete, or incorrectly coded, and should be interpreted with caution. It is therefore recommended that all adverse events [AEs] possibly related to the study interventions be collected carefully and with accurate timing, for the entire duration of the observation, even after the end of the study treatment. Serious adverse events and adverse events leading to treatment discontinuation deserve special attention and should be analysed separately, as their impact on patients is critical.

#### 4.3.8. Statement 21 [7.7]

##### If relevant to an intervention, sample size should be adequate to detect uncommon adverse effects of interest.

In studies primarily addressing safety, the sample size should be large enough to detect also relevant uncommon adverse events. This is crucial in order not to underestimate the relevance of uncommon side effects because of inadequate sample size.

### 4.4. Topic 4—Analysis

#### 4.4.1. Statement 22 [8.6]

##### As the intention-to-treat effect is the effect of interest in pragmatic trials, the primary analysis should be performed according to the intention-to-treat approach. A per-protocol analysis may be considered as part of secondary analyses.

Although there is no standard definition of intention-to-treat [ITT], the American Statistical Association [ASA] gave what is probably the most widely accepted version:

‘An intention to treat analysis is one which includes all randomised patients in the groups to which they were randomly assigned, regardless of the compliance with the entry criteria, regardless of the treatment they actually received, and regardless of subsequent withdrawal from treatment or deviation from the protocol.’^47^

In a pragmatic trial it is neither necessary nor always desirable for all subjects to complete the trial in the group to which they were allocated. However, patients are always analysed in the group to which they were initially randomised [intention-to-treat analysis], even if they drop out of the study or change groups. The intention-to-treat approach reduces the possibility of overestimating any clinical effectiveness and is therefore most suitable for pragmatic RCTs.

On the other hand, the per-protocol effect is what would have been observed if all patients had adhered to the trial protocol. In other words, the per-protocol effect is not affected by incomplete adherence and may therefore be of greater interest to patients who are considering whether to use the treatment. Non-inferiority studies are analysed per-protocol. Proper per-protocol analysis requires adjustments for confounding due to incomplete adherence. Therefore, per-protocol analyses should remain secondary analyses.^48^

#### 4.4.2. Statement 23 [8.6]

##### In a pragmatic trial, the planned methods to deal with missing data should be prespecified in the protocol.

A major challenge when using routinely collected data is to produce valid and accurate results. As data collection occurs under real-life conditions, higher levels of missing data and entry errors may be expected, possibly resulting in biases particularly, but not exclusively, when measurement error does not occur randomly. Furthermore, random errors in data collection and missing data can reduce the power of the study, with implications for the calculation of the required sample size. Missing data may be more prevalent in a pragmatic trial [given its less restrictive protocol] than in an explanatory trial, in which monitoring is more stringent. Therefore, the planned methods to deal with missing data should be prespecified in the protocol. Maximum missing data permissible should also be statistically specified. Mitigation strategies to minimise missing data should be adopted, including acceptance of rescue interventions, flexible data collection, enhancement of engagement in study, and reduction of study burden.

#### 4.4.3. Statement 24 [8.0]

##### Complex statistical methods to handle missing data, such as multiple imputation or covariate adjustment, are to be preferred to single imputation methods.

In explanatory trials, the use of standard [single] imputation techniques (such as ‘last observation carried forward’ [LOCF] or ‘non-responder imputation [NRI]) could be justified by the fact that, in accordance with the protocol, the patient would be required to continue receiving the study treatment. In a pragmatic trial, this should usually not be the case, as changes to treatment and drop-out are likely to occur more often. Discontinuation of intervention should be distinguished from study withdrawal/drop-outs. Thus in pragmatic trials, standard [single] imputation techniques, and specially LOCF, are not recommended to deal with missing data, whereas more complex statistical methods, such as multiple imputation or covariate adjustment, should be preferred.

In particular the LOCF method, in which missing final values of the outcome variable are replaced by the last known value before the participant was lost to follow-up, is widely used. This method may seem appealing due to its simplicity, but it may introduce bias and no allowance is made for the uncertainty of imputation. Therefore many authors have severely criticised LOCF and currently recommend that it should be used with caution. On the other hand, NRI is a common statistical approach for the analysis of binary efficacy variables. According to the NRI rule, all values missing for any, reason, including discontinuation from study or switching to rescue medications, are considered as ‘not achieved’. NRI analyses tend to result in more conservative estimates of drug effect on outcome measures than LOCF analyses, particularly in trials with a high number of participant drop-outs.^49^

In conclusion, single imputation methods are not recommended in pragmatic trials. However in studies including patients with IBD, where most of the missing data are due to lack of response to treatment [primary non-response or loss of response], NRI, providing less conservative [i.e. negative] estimate, should be preferred to LOCF.

#### 4.4.4. Statement 25 [8.4]

##### The emulation of a hypothetical target trial using non-randomised real-world data reduces the risk of self-inflicted bias. Appropriate propensity score methods may achieve balance between groups on collected variables.

Attempting to reduce the risk of bias in studies based on non-randomised, real-world data requires both methodological and clinical expertise. The participation of both methodologists with experience in the relevant study designs, and health professionals with knowledge of prognostic factors that influence intervention decisions for the target population, is recommended. At the planning stage, the review question must be clearly articulated, specifying important confounders and co-interventions. Designing the analysis to explicitly emulate a [hypothetical] target trial, whose protocol component should be similar to those of RCTs [i.e. eligibility criteria, outcome, treatment strategies, start/end of follow-up, causal contrast, and statistical analysis], is advisable to prevent bias related to study design, such as immortal time bias.^50^

Propensity score [PS] methods have been widely used to reduce confounding biases in observational studies. The PS is the probability that a patient would receive the treatment of interest, based on collected variables included in the PS model. After estimation, confounding adjustment through conditioning on the PS can be done by various methods, including matching and weighting. It should be emphasised that balance between treatment groups is assessable using standardised mean differences only for covariates included in the PS model. Using fit-for-purpose data, PS methods may enable clinical researchers to obtain balanced treatment groups similar to an RCT.^35,36^

Statement application and data reporting: far from being ‘essential’ elements of a pragmatic trial, the proposed statements offer a guide in trial design and they may be considered in the phase of data reporting. To facilitate both these phases, we have elaborated a check list intended to provide a dry summary of all concepts incorporated in each consensus’s statement [Supplementary Figure S2] and by a yes-or-no response to indicate to the user how far a study is from the proposed model of pragmatic trial. We also encourage use of the checklist as a guide to indicate in the method section why and how a reported study can be considered pragmatic based on this consensus’s statements.

## 5. Conclusions

pRCTs can effectively complement the evidence from registration and explanatory trials, and they should be encouraged to compare different treatment strategies in IBD. Pragmatic trials should represent a step forward defining effectiveness in a real-world setting. Therefore, the proposed recommendations mainly apply to Phase 4 studies. However, we cannot exclude that a pragmatic study design could be applied to Phase 3 and even Phase 2 studies to generate efficacy and effectiveness data jointly, particularly when comparing new yet not approved therapies with others already on the market and used in clinical practice. Moreover, pharmaceutical companies could adopt a more pragmatic approach during early phases of development when the demonstration of effectiveness in a real-world setting, in addition to RCT efficacy data, may lead to a faster approval of the product [i.e. fast track] as suggested by Wallach JD *et al*.^51^ Pragmatism should not be synonymous with a lax and non-methodologically rigorous approach to trial conduct. Since the goal is to properly inform clinical practice, this can only be achieved with high-quality studies. It is worth mentioning that during the discussion no specific features emerged differentiating pragmatic trials in IBD from those of other areas. However, we believe that some aspects, such as how to manage concomitant medications, the choice of specific outcomes, and the definition of minimal inclusion criteria, are specific and should be well defined when designing pragmatic trials in the IBD field. The best approach is to design a study that can adequately answer the clinical questions it poses and meet the intended users’ needs. These statements, produced by a European group of experts in IBD and trial methodology, intend to offer practical guidance in the design of pragmatic clinical trials that can aid decision making in choosing a management strategy for IBDs.

The PRACTICE-IBD study group

Matthieu Allez, Service de Gastroentérologie, Hôpital Saint-Louis, APHP, Université Paris Cité, Paris, France. Aurelien Amiot, Department of Gastroenterology, Bicêtre University Hospital, AP-HP, Paris-Saclay University, Le Kremlin Bicêtre, France. Raja Atreya, Medical Department 1, University Hospital Erlangen, Friedrich-Alexander-University of Erlangen-Nürnberg, Ulmenweg, Erlangen, Germany. Manuel Barreiro-de Acosta, Gastroenterology Department of Complexo Hospitalario Universitario de Santiago, Santiago de Compostela, A Coruña, Spain. Livia Biancone, Department of Systems Medicine, University “Tor Vergata” of Rome, Rome, Italy. Fabiana Castiglione, Gastroenterology, Department of Clinical Medicine and Surgery, University Federico II of Naples, Naples, Italy. María Chaparro, Gastroenterology Unit, Hospital Universitario de La Princesa, Instituto de Investigación Sanitaria Princesa [IIS-Princesa], Universidad Autónoma de Madrid [UAM], Centro de Investigación Biomédica en Red de Enfermedades Hepáticas y Digestivas [CIBEREHD], Madrid, Spain. Axel Dignass, Department of Medicine I, Agaplesion Markus Hospital, Goethe University, Frankfurt, Germany. Glen Doherty, Department of Gastroenterology, St Vincent’s University Hospital and School of Medicine, University College Dublin, Ireland. Eugeni Domènech, Gastroenterology Department, Hospital Universitari Germans Trias i Pujol, Badalona; Centro de Investigación Biomédica en Red de Enfermedades Hepáticas y Digestivas—CIBEREHD; Departament de Medicina, Universitat Autònoma de Barcelona [Spain]. Walter Fries, IBD-Unit, Department of Clinical and Experimental Medicine, University of Messina, Messina, Italy. Jonas Halfvarson, Department of Gastroenterology, Faculty of Medicine and Health, Örebro University, Örebro, Sweden. Uri Kopylov, Sheba Medical Center, Ramat Gan and Tel Aviv University Medical School, Tel Aviv Israel. Sara Onali, Department of Medical Science and Public Health, Gastroenterology Unit, University of Cagliari, Cagliari, Italy; Gastroenterology Unit, Azienda Ospedaliero-Universitaria [AOU] di Cagliari, Cagliari, Italy. Daniela Pugliese, CEMAD, IBD Unit, Dipartimento di Scienze Mediche e Chirurgiche, Fondazione Policlinico Universitario “A. Gemelli” IRCCS, Università Cattolica del Sacro Cuore, Rome, Italy. Davide Giuseppe Ribaldone, Dipartimento di Scienze Mediche, Università degli Studi di Torino, Turin, Italy. Simone Saibeni, Gastroenterology Unit, Rho Hospital, ASST Rhodense, Rho [MI], Italy. Edoardo Vincenzo Savarino, Department of Surgery, Oncology and Gastroenterology, University of Padova, Padova, Italy.

## Supplementary Data

Supplementary data are available at *ECCO-JCC* online.

jjae026_suppl_Supplementary_Figure_S1

jjae026_suppl_Supplementary_Figure_S2

jjae026_suppl_Supplementary_Material

jjae026_suppl_Supplementary_Table_S1

jjae026_suppl_Supplementary_Table_S2

## Data Availability

The data underlying this article are available in the article and in its online supplementary material
