# Supplementary figures and images for: Pragmatic Trial Design to Compare Real-world Effectiveness of Different Treatments for Inflammatory Bowel Diseases: The PRACTICE-IBD European Consensus
Source: J Crohns Colitis. 2024 Feb 17;18(8):1222–31. doi: 10.1093/ecco-jcc/jjae026 (PMC11324339; doi:10.1093/ecco-jcc/jjae026)

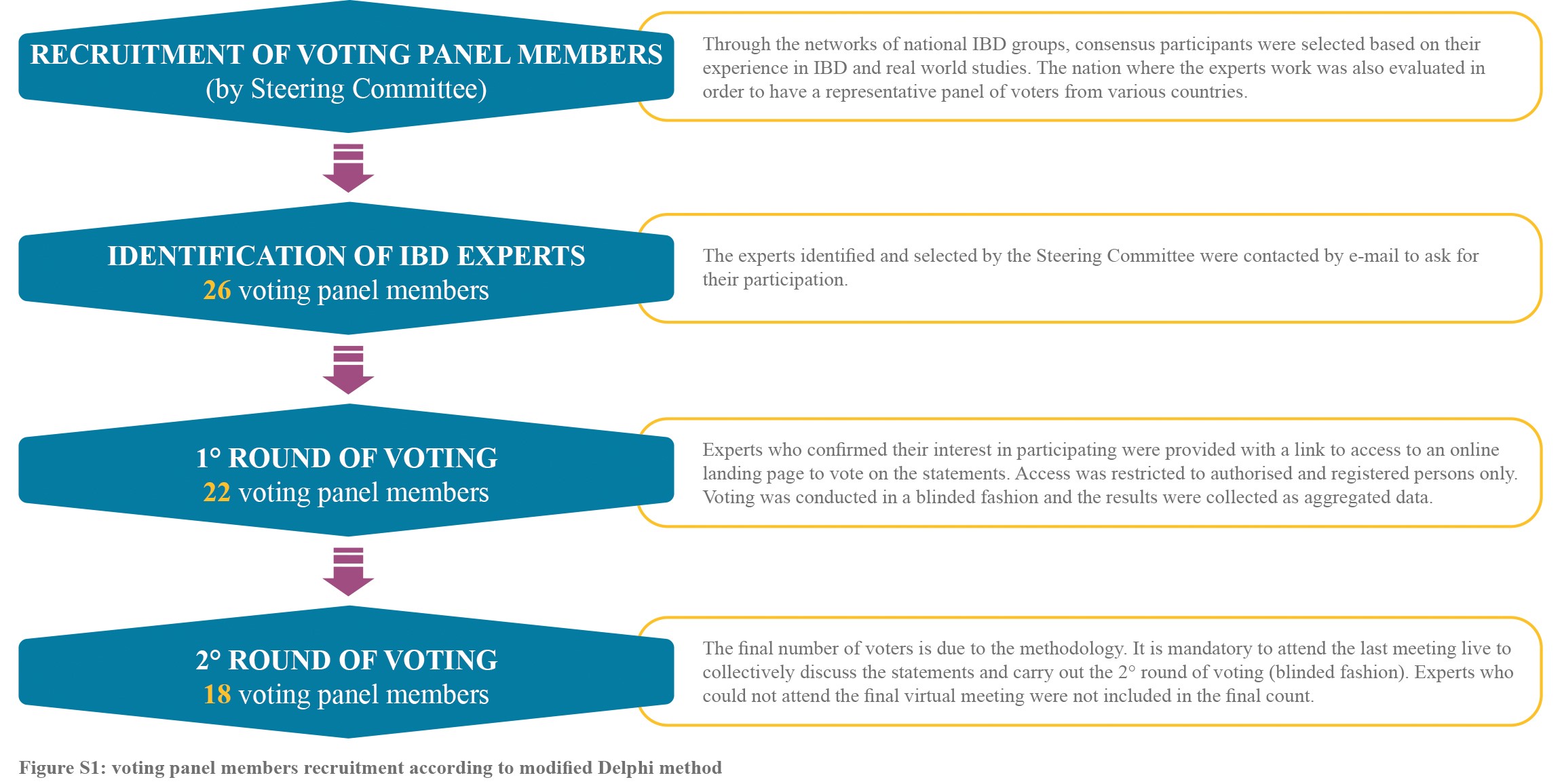

Supplement: jjae026_suppl_Supplementary_Figure_S1 [file jjae026_suppl_supplementary_figure_s1.jpeg]

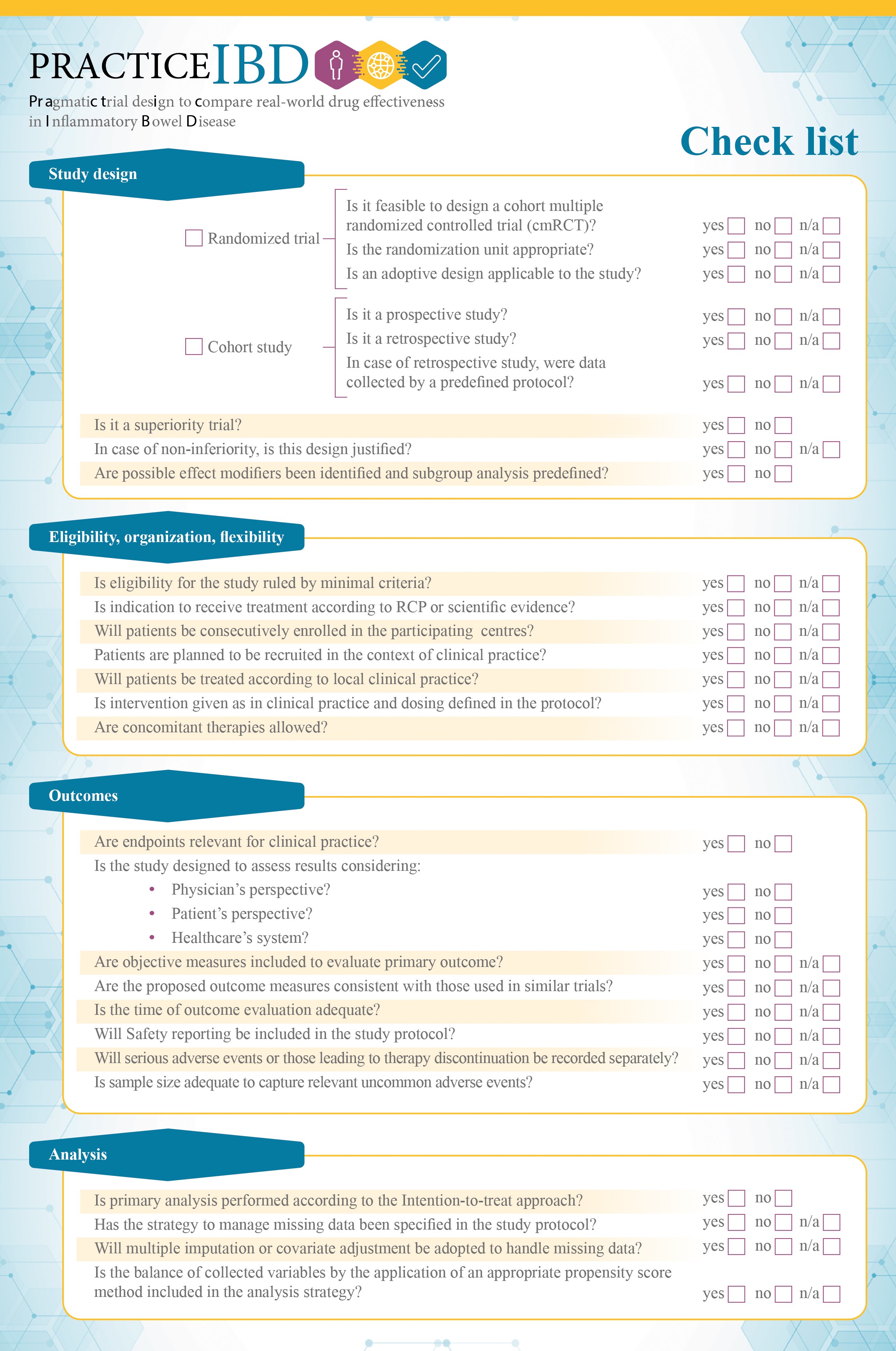

Supplement: jjae026_suppl_Supplementary_Figure_S2 [file jjae026_suppl_supplementary_figure_s2.jpeg]
