# Supplementary material for: Pragmatic Trial Design to Compare Real-world Effectiveness of Different Treatments for Inflammatory Bowel Diseases: The PRACTICE-IBD European Consensus
Source: J Crohns Colitis. 2024 Feb 17;18(8):1222–31. doi: 10.1093/ecco-jcc/jjae026 (PMC11324339; doi:10.1093/ecco-jcc/jjae026)
Supplement: jjae026_suppl_Supplementary_Material [file jjae026_suppl_supplementary_material.docx]

**Supplementary Methods**

**Search strategy**

The initial search was conducted by members of the steering committee. The search strategies were first developed in MEDLINE and subsequently adapted to other bibliographic databases (e.g. EMBASE, Google Scholar, Web of Science, SCOPUS). The search terms included subject headings (e.g., MeSH) specific to each database and free-text words relevant to pragmatic trials. We screened reference lists of relevant publications, including reviews and meta-analyses, for relevant articles. We used the following search terms: “Pragmatic AND trial AND (“Crohn’s” AND “ulcerative colitis”) AND drugs”– tot. 3 results; “Pragmatic AND trial AND (“Inflammatory Bowel Disease” OR “IBD”) AND drugs” – tot. 5 results; “("Crohn's" OR "Ulcerative colitis") AND pragmatic AND trial” – tot. 37 results; “("Inflammatory bowel disease" OR "IBD") AND pragmatic AND trial” – tot. 39 results. After discarding duplicates, a total number of 39 publications were examined and only two of the examined papers reported on declared pragmatic trials [^1-3^](#_ENREF_1). Due to the exiguity number of pragmatic trials in the IBD field we decided to proceed with a narrative literature review approach starting from the analysis of two Delphi consensuses on pragmatic studies the PRECIS and PRECIS-2 consensuses [^4-6^](#_ENREF_4). From the publication references listed in these two papers and from a comprehensive search of the available literature outside the IBD filed (search terms: “Pragmatic trials” AND “comparative”; “Pragmatic trials” AND “drug”; “Pragmatic trials” AND “therapy”; “Pragmatic trials” AND review) papers considered relevant for the object of the consensus were made available to all members of the steering committee and scientific board (**Table S1**).

**References:**

1 Seagrove AC, Alam MF, Alrubaiy L, Cheung WY, Clement C, Cohen D, Grey M, Hilton M, Hutchings H, Morgan J, Rapport F, Roberts SE, Russell D, Russell I, Thomas L, Thorne K, Watkins A, Williams JG. Randomised controlled trial. Comparison Of iNfliximab and ciclosporin in STeroid Resistant Ulcerative Colitis: Trial design and protocol (CONSTRUCT). *BMJ open*. 2014;4(4):e005091.

2 Smits LJT, Pauwels RWM, Kievit W, de Jong DJ, de Vries AC, Hoentjen F, van der Woude CJ, group Ls. Lengthening adalimumab dosing interval in quiescent Crohn's disease patients: protocol for the pragmatic randomised non-inferiority LADI study. *BMJ open*. 2020;10(5):e035326.

3 Williams JG, Alam MF, Alrubaiy L, Clement C, Cohen D, Grey M, Hilton M, Hutchings HA, Longo M, Morgan JM, Rapport FL, Seagrove AC, Watkins A. Comparison Of iNfliximab and ciclosporin in STeroid Resistant Ulcerative Colitis: pragmatic randomised Trial and economic evaluation (CONSTRUCT). *Health technology assessment*. 2016;20(44):1-320.

4 Loudon K, Treweek S, Sullivan F, Donnan P, Thorpe KE, Zwarenstein M. The PRECIS-2 tool: designing trials that are fit for purpose. *Bmj*. 2015;350:h2147.

5 Loudon K, Zwarenstein M, Sullivan F, Donnan P, Treweek S. Making clinical trials more relevant: improving and validating the PRECIS tool for matching trial design decisions to trial purpose. *Trials*. 2013;14:115.

6 Thorpe KE, Zwarenstein M, Oxman AD, Treweek S, Furberg CD, Altman DG, Tunis S, Bergel E, Harvey I, Magid DJ, Chalkidou K. A pragmatic-explanatory continuum indicator summary (PRECIS): a tool to help trial designers. *Journal of clinical epidemiology*. 2009;62(5):464-475.
