# Supplementary material for: Pragmatic Trial Design to Compare Real-world Effectiveness of Different Treatments for Inflammatory Bowel Diseases: The PRACTICE-IBD European Consensus
Source: J Crohns Colitis. 2024 Feb 17;18(8):1222–31. doi: 10.1093/ecco-jcc/jjae026 (PMC11324339; doi:10.1093/ecco-jcc/jjae026)
Supplement: jjae026_suppl_Supplementary_Table_S1 [file jjae026_suppl_supplementary_table_s1.docx]

**Table S1. Articles considered relevant for the consensus made available to all members of the steering committee and scientific board**

| **#** | **References** |
| --- | --- |
| 1 | Anstrom, K.J., et al., Design and rationale of a multi-center, pragmatic, open-label randomized trial of antimicrobial therapy - the study of clinical efficacy of antimicrobial therapy strategy using pragmatic design in Idiopathic Pulmonary Fibrosis (CleanUP-IPF) clinical trial. Respir Res, 2020. 21(1): p. 68. |
| 2 | Choudhry, N.K., et al., *Full coverage for preventive medications after myocardial infarction.* N Engl J Med, 2011. **365**(22): p. 2088-97. |
| 3 | Davies-Teye, B.B., et al., *Pragmatic patient engagement in designing pragmatic oncology clinical trials.* Future Oncol, 2021. **17**(28): p. 3691-3704. |
| 4 | Ford, I. and J. Norrie, *Pragmatic Trials.* N Engl J Med, 2016. **375**(5): p. 454-63. |
| 5 | Frobert, O., et al., *Thrombus aspiration during ST-segment elevation myocardial infarction.* N Engl J Med, 2013. **369**(17): p. 1587-97. |
| 6 | Kauf, T.L., et al., *An open-label, pragmatic, randomized controlled clinical trial to evaluate the comparative effectiveness of daptomycin versus vancomycin for the treatment of complicated skin and skin structure infection.* BMC Infect Dis, 2015. **15**: p. 503. |
| 7 | Loudon, K., et al., *The PRECIS-2 tool: designing trials that are fit for purpose.* BMJ, 2015. **350**: p. h2147. |
| 8 | Loudon, K., et al., *Making clinical trials more relevant: improving and validating the PRECIS tool for matching trial design decisions to trial purpose.* Trials, 2013. **14**: p. 115. |
| 9 | McMahon, A.D., *Study control, violators, inclusion criteria and defining explanatory and pragmatic trials.* Stat Med, 2002. **21**(10): p. 1365-76. |
| 10 | Ostuzzi, G., et al., *Tolerability and efficacy of vortioxetine versus SSRIs in elderly with major depression. Study protocol of the VESPA study: a pragmatic, multicentre, open-label, parallel-group, superiority, randomized trial.* Trials, 2020. **21**(1): p. 695. |
| 11 | Patsopoulos, N.A., *A pragmatic view on pragmatic trials.* Dialogues Clin Neurosci, 2011. **13**(2): p. 217-24. |
| 12 | Pawson, R., *The shrinking scope of pragmatic trials: a methodological reflection on their domain of applicability.* J Clin Epidemiol, 2019. **107**: p. 71-76. |
| 13 | Qu, H., S. Austin, and J.A. Singh, *Identifying physician-perceived barriers to a pragmatic treatment trial in rheumatoid arthritis.* Eur J Rheumatol, 2022. **9**(3): p. 132-138 |
| 14 | Ridd, M.J., et al., *Effectiveness and safety of lotion, cream, gel, and ointment emollients for childhood eczema: a pragmatic, randomised, phase 4, superiority trial.* Lancet Child Adolesc Health, 2022. **6**(8): p. 522-532. |
| 15 | Roberts, I., et al., *Effect of intravenous corticosteroids on death within 14 days in 10008 adults with clinically significant head injury (MRC CRASH trial): randomised placebo-controlled trial.* Lancet, 2004. **364**(9442): p. 1321-8. |
| 16 | Schwartz, D. and J. Lellouch, *Explanatory and pragmatic attitudes in therapeutical trials.* J Clin Epidemiol, 2009. **62**(5): p. 499-505. |
| 17 | Seagrove, A.C., et al., *Randomised controlled trial. Comparison Of iNfliximab and ciclosporin in STeroid Resistant Ulcerative Colitis: Trial design and protocol (CONSTRUCT).* BMJ Open, 2014. **4**(4): p. e005091. |
| 18 | Smits, L.J.T., et al., *Lengthening adalimumab dosing interval in quiescent Crohn's disease patients: protocol for the pragmatic randomised non-inferiority LADI study.* BMJ Open, 2020. **10**(5): p. e035326. |
| 19 | Thorpe, K.E., et al., *A pragmatic-explanatory continuum indicator summary (PRECIS): a tool to help trial designers.* J Clin Epidemiol, 2009. **62**(5): p. 464-75. |
| 20 | Treweek, S. and M. Zwarenstein, *Making trials matter: pragmatic and explanatory trials and the problem of applicability.* Trials, 2009. **10**: p. 37. |
| 21 | van der Leeuw, M.S., et al., *Effectiveness of TOcilizumab in comparison to Prednisone In Rheumatoid Arthritis patients with insufficient response to disease-modifying antirheumatic drugs (TOPIRA): study protocol for a pragmatic trial.* Trials, 2020. **21**(1): p. 313. |
| 22 | Wallach, J.D., et al., *Feasibility of Using Real-world Data to Emulate Postapproval Confirmatory Clinical Trials of Therapeutic Agents Granted US Food and Drug Administration Accelerated Approval.* JAMA Netw Open, 2021. **4**(11): p. e2133667. |
| 23 | Williams, J.G., et al., *Comparison Of iNfliximab and ciclosporin in STeroid Resistant Ulcerative Colitis: pragmatic randomised Trial and economic evaluation (CONSTRUCT).* Health Technol Assess, 2016. **20**(44): p. 1-320. |
