# Supplementary material for: Pragmatic Trial Design to Compare Real-world Effectiveness of Different Treatments for Inflammatory Bowel Diseases: The PRACTICE-IBD European Consensus
Source: J Crohns Colitis. 2024 Feb 17;18(8):1222–31. doi: 10.1093/ecco-jcc/jjae026 (PMC11324339; doi:10.1093/ecco-jcc/jjae026)
Supplement: jjae026_suppl_Supplementary_Table_S2 [file jjae026_suppl_supplementary_table_s2.docx]

**Table S2. Consensus statements on how to design pragmatic trials in inflammatory bowel diseases (IBDs).**

| **Statement** | 1^st^ round  rating  (mean value ± SD) | **2^nd^ round rating**  **(mean value** ± SD**)** |
| --- | --- | --- |
| 1. ***Randomization should be considered as the first option to assess and compare the effect of interventions in a pragmatic trial.*** | 8.3 ± 0.7 | ***8.6*** ± 0.5 |
| 1. The unit of randomization and intervention can not only be patients, but also healthcare practitioners, communities, or healthcare institutions such as clinics depending on the nature of the intervention, setting and the aim of the study.   Revised as:  ***The unit of randomization and analysis may be patients, but also healthcare practitioners, communities, or healthcare institutions such as clinics, depending on the nature of the intervention, setting and aim of the study.*** | 7.7 ± 1.0 | ***8.2*** ± 0.6 |
| 1. Cohort multiple randomized controlled trials (cmRCTs) allow to recruit and retain patients more effectively and make trials more efficient and patient centered.   Revised as:  ***Cohort multiple randomized controlled trials (cmRCTs) allow to recruit and retain patients*** ***effectively and make trials efficient and patient centered****.* | 7.4 ± 1.0 | ***8.5*** ± 0.6 |
| 1. ***Adaptive trial design may increase efficiency and make trials more conclusive, especially if multiple interventions are compared at the same time.*** | 7.6 ± 1.1 | ***8.3*** ± 0.8 |
| 1. Longitudinal (follow-up) cohort studies can properly use a retrospective or prospective design.   Revised as:  ***Longitudinal (follow-up) cohort studies should be***  ***preferably prospective but can also use a***  ***retrospective design.*** | 7.0 ± 1.3 | ***8.1*** ± 0.7 |
| 1. In order to identify possible moderators of effects, planning prespecified subgroup analyses is needed.   Revised as:  ***To identify possible modifiers of effects, planning prespecified subgroup analyses is*** ***recommended****.* | 7.9 ± 0.8 | ***8.2*** ± 1.8 |
| 1. Noninferiority trials may be justified if the investigated therapy is cheaper, more convenient, less invasive, or less toxic than the active control.   Revised as:  ***Noninferiority trials may be justified if the investigated therapy is cheaper, more convenient, or safer than the control therapy.*** | 7.5 ± 1.4 | ***8.4*** ± 0.6 |
| 1. ***Minimal criteria for eligibility in pragmatic studies aiming to compare drug effectiveness should include confirmed diagnosis and indication to receive the drugs under investigation according to Summary of Product Characteristics (SmPC) or robust scientific evidence*.** | 8.1 ± 1.0 | ***8.4*** ± 0.8 |
| 1. ***In prospective studies, consecutive patients with the diagnosis of interest and indication for the treatment under study accessing participating centers should be invited to participate.*** | 8.1 ± 1.1 | ***8.4*** ± 0.8 |
| 1. *Recruitment should be performed in the context of usual care.*   Revised as:  ***Recruitment should be performed in the context of standard clinical practice*** | 8.1 ± 1.0 | ***8.8*** ± 0.6 |
| 1. ***Patients should be treated in a real-world setting, according to local clinical practice.*** | 8.0 ± 1.1 | ***8.6*** ± 0.7 |
| 1. The intervention should be given as used in clinical practice and any possible dose should be clearly defined in the study protocol and rigorously reported.   Revised as:  ***The intervention should be given as in clinical practice and dosing regimen should be clearly defined in the study protocol and rigorously reported.*** | 8.1 ± 1.0 | ***8.7*** ± 0.5 |
| 1. To score highly pragmatic there should be no or minimal restrictions on concomitant therapies.   Revised as:  ***A pragmatic study design should include minimal restrictions on concomitant therapies to reflect practical setting.*** | 7.0 ± 1.7 | ***8.7*** ± 0.6 |
| 1. ***In pragmatic studies comparing drugs or strategies, effectiveness can be defined as the capability of any intervention to achieve predetermined endpoints relevant for producing a desired result in clinical practice.*** | 8.0 ± 1.0 | ***8.7*** ± 0.5 |
| 1. Depending on the question of interest, effectiveness should be assessed considering the perspectives of the physician, the patient and/or the healthcare system.   Revised as:  ***Depending on the question of interest, the results should***  ***be assessed considering the perspectives of the physician,***  ***the patient and/or the healthcare system.*** | 7.3 ± 1.3 | ***8.1*** ± 1.7 |
| 1. Objective measures of effectiveness should be preferred to assess the primary outcome.   Revised as:  ***Objective established measures of effectiveness should be preferred to assess the primary outcome.*** | 8.1 ± 1.0 | ***8.5*** ± 0.5 |
| 1. A pragmatic trial should have one primary endpoint. A composite primary endpoint may also be considered.   Revised as:  ***A pragmatic trial should have only one primary endpoint. A composite primary endpoint may also be considered.*** | 7.9 ± 1.2 | ***8.7*** ± 0.6 |
| 1. Outcome measures should always be clearly listed in a pragmatic trial; they should be consistent, as much as possible, with outcome measures used in similar trials, if any.   Revised as:  ***Outcome measures should always be clearly defined in a pragmatic trial; they should be consistent, as much as possible, with those used in similar trials.*** | 8.0 ± 1.0 | ***8.6*** ± 0.5 |
| Study outcomes strongly dependent on the subjective investigator’s judgement should be avoided, unless based on standard scales/scores.  **DELETED** | 7.6 ± 1.4 |  |
| 1. Effectiveness should be assessed after an adequate time interval depending on the drug/strategy investigated. Short-term response should be assessed at the latest time a drug is expected to exert its therapeutic effect. Long-term outcomes should be assessed not earlier than 52 weeks.   Revised as:  ***Outcomes should be assessed after an adequate time interval, depending on the drug/strategy investigated.*** | 7.8 ± 1.1 | ***8.4*** ± 0.7 |
| 1. Safety should be assessed considering any adverse event possibly related to the study drug. Serious adverse events and adverse events leading to discontinuation should always be recorded and analysed separately. In studies primarily addressed on safety, the sample size should be adequate to detect possible uncommon relevant adverse events.   SPLIT INTO:  ***Safety should be assessed considering any adverse event possibly related to the study drug/intervention. Serious adverse events and adverse events leading to discontinuation should always be recorded and analysed separately.***   1. ***If relevant to an intervention, sample size should be adequate to detect uncommon adverse effects of interest.*** | 8.2 ± 1.0 | ***8.0*** ± 1.9  ***7.7*** ± 2.4 |
| 1. As the intention-to-treat effect is the effect of interest in pragmatic trials, the primary analysis should be performed according to the intention-to-treat approach, which reduces the possibility of overestimating any clinical effectiveness. Additional analyses, such as a per-protocol analysis, may therefore be considered as secondary analyses.   Revised as:  ***As the intention-to-treat effect is the effect of interest in*** ***pragmatic trials, the primary analysis should be performed according to the intention-to-treat approach****.* ***A per-protocol analysis may be considered as part of secondary analyses.*** | 7.9 ± 0.9 | ***8.6*** ± 0.7 |
| 1. Missing data may be more prevalent in a pragmatic trial (given its less restrictive protocol) than in explanatory trials, in which monitoring is more stringent. The planned methods to deal with missing data should be prespecified in the protocol. Maximum missing data permissible should be statistically specified.   Revised as:  ***In a pragmatic trial the planned methods to deal with missing data should be prespecified in the protocol.*** | 7.8 ± 1.1 | ***8.6*** ± 0.5 |
| 1. Complex statistical methods to handle missing data, such as multiple imputation or covariate adjustment, are to be preferred to Non-Responder Imputation (NRI) analysis.   Revised as:  ***Complex statistical methods to handle missing data, such as multiple imputation or covariate adjustment, are to be preferred*** ***to single imputation methods****.* | 6.9 ± 1.7 | ***8.0*** ± 1.2 |
| The last observation carried forward (LOCF) method is considered not appropriate to handle missing data in pragmatic trials.  **DELETED** | 7.2 ± 1.0 |  |
| 1. The emulation of a hypothetical target trial using nonrandomized real-world data reduces the risk of self-inflicted bias. Appropriate propensity score methods with expert statistical input may achieve balance between treatment groups on collected variables.   Revised as:  ***The emulation of a hypothetical target trial using nonrandomized real-world data reduces the risk of self-inflicted bias. Appropriate propensity score methods may achieve balance between groups on collected variables.*** | 7.0 ± 1.1 | ***8.4*** ± 0.7 |
